# Case Report: Novel Copy Number Variant 16p11.2 Duplication Associated With Prune Belly Syndrome

**DOI:** 10.3389/fped.2021.729932

**Published:** 2021-09-23

**Authors:** Sriharsha Talluri, Michael A. Goedde, Eran Rosenberg, Katie L. Canalichio, Dennis Peppas, Jeffrey T. White

**Affiliations:** ^1^Department of Urology, University of Louisville, Louisville, KY, United States; ^2^Department of Pediatric Urology, Norton Healthcare, Louisville, KY, United States

**Keywords:** prune belly syndrome, pediatric urology, chromosomal duplication, cryptorchidism, vesicoureteral reflux

## Abstract

Prune belly syndrome (PBS) is a rare congenital disease that predominantly occurs in males and is identified by its classic triad of abdominal wall musculature deficiencies, cryptorchidism, and urinary tract abnormalities. However, numerous anomalies involving the kidneys, heart, lungs, and muscles have also been reported. A multitude of chromosomal abnormalities have been implicated in its pathogenesis. PBS can occur in association with trisomy 18 and 21. Gene duplications and deletions have also been reported; however, a definite cause of PBS is still unknown. We report the first PBS patient with a copy number variant in 16p11.2.

## Introduction

Prune belly syndrome (PBS) is a rare congenital disease occurring predominantly in males. It has also been referred to as Eagle-Barrett syndrome, triad syndrome, and Obrinsky's syndrome and is known by its classic triad of abdominal wall musculature deficiencies, cryptorchidism, and urinary tract abnormalities (1). Despite numerous case reports and reviews, there is a scarcity of information in the literature regarding definitive causes. Several theories have been surmised, including primary defect of intermediate and lateral plate mesoderm (2, 3), intrinsic defect of the urinary tract, and *in utero* urethral obstruction (4). While chromosomal abnormalities do occur in PBS, it is the exception, not the rule. We report a male patient with PBS and a 16p11.2 chromosomal duplication.

## Case Report

**Figure 1:**
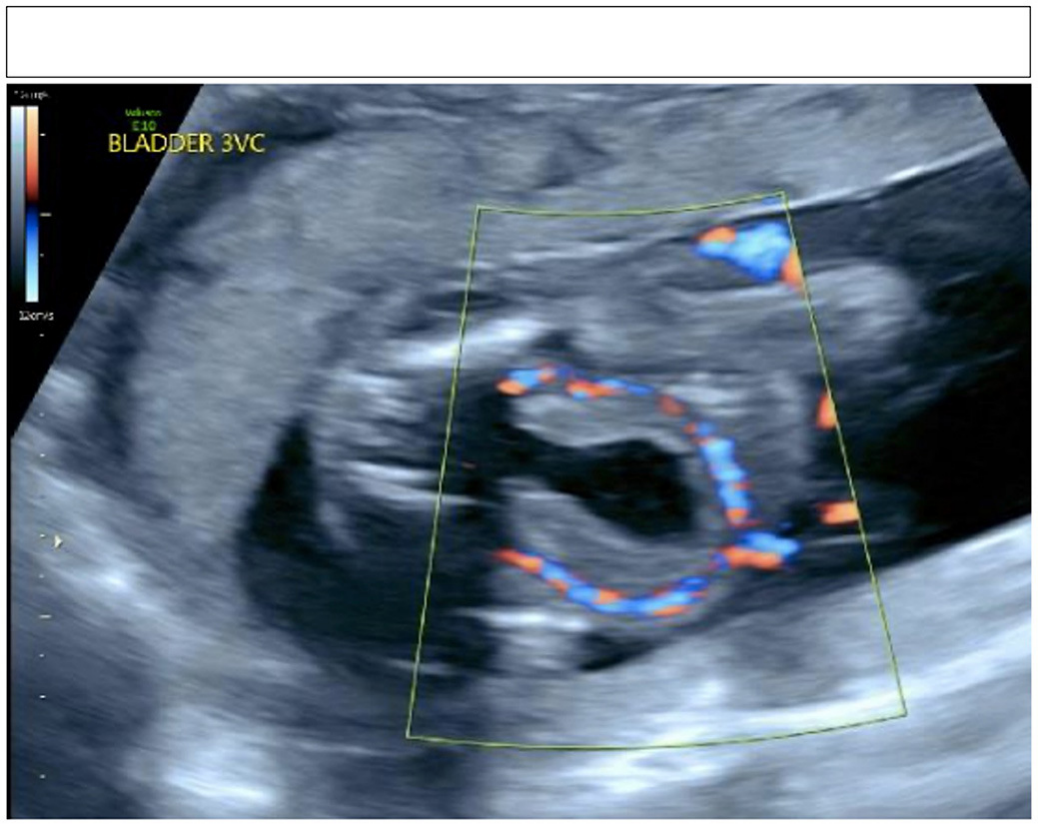
Keyhole sign on prenatal ultrasonography.

Urology was consulted for newborn male with urinary dribbling accompanied with a distended abdomen. He was born *via* repeat cesarean section prematurely at 35 weeks 3 days' gestation. The pregnancy was complicated by breech presentation and oligohydramnios. Prenatal ultrasound at 26 weeks showed dilated bilateral upper urinary tracts, thick-walled distended bladder with a “keyhole sign,” and dilated posterior and anterior urethra (see Figure 1). Upon delivery, he was intubated in the delivery room for respiratory distress, and placement of a urinary catheter was unsuccessful.

On examination, the patient's abdomen was distended with reduced abdominal wall musculature. There was tight phimosis of an uncircumcised phallus, and his scrotum was underdeveloped without palpable testes bilaterally.

Urinary catheterization was successful by urology with a six French Foley catheter. Shortly after birth, his creatinine was 0.5 mg/dl. He was extubated overnight on day of life (DOL) 1. Due to concern for urinary tract infection, the patient was started on antibiotic prophylaxis. His creatinine rose to a peak of 1.9 mg/dl and was trended until it reached a level of 1.1 mg/dl on DOL 16. It was not until 2 months of life that creatinine reached a nadir at 0.5 mg/dl. Renal ultrasound showed small kidneys with multiple cysts and massive bilateral hydroureter with mild hydronephrosis. Voiding cystourethrogram revealed absence of vesicoureteral reflux (VUR), poor emptying, and no apparent urethral obstruction.

Due to the presence of bilateral undescended testes, upper tract dilation, and abdominal abnormality, a diagnosis of incomplete PBS (Woodard classification 2/3) was made. Prior to discharge on DOL 16, the patient was started on clean intermittent catheterization.

## Discussion

Though the incidence of PBS has been estimated to be ~3.8 per 100,000 live births (5), case reports and reviews of this rare disease are quite numerous. Much of the literature has shown that there is a higher incidence among the African American population (5, 6). Additionally, an infrequent presentation without cryptorchidism has been seen in females (6–8).

The presentation of those with PBS can be quite variable. The abdominal exam can range from mildly protuberant without “pruning” to grossly thin and wrinkled abdominal wall (9). Observed urinary tract abnormalities include VUR, hydroureteronephrosis, hypospadias, and patent urachus (6). Renal dysplasia occurs in roughly 5% of patients. VUR occurs in 75% of patients accompanied with immense ureteral dilation as stroma replaces smooth muscle. The bladder is massively enlarged with properties of delayed sensation but usually normal compliance. The posterior urethra is dilated due to prostatic hypoplasia, while the anterior urethra can range from atretic to a megalourethra. Aside from this classic triad of PBS, multiple sources have reported numerous additional features that occurred in patients with PBS, some of which include chronic kidney disease, pulmonary hypoplasia, cardiac anomalies, musculoskeletal anomalies, constipation, and malrotation (6, 9–12). Though the Woodard classification system was initially developed to stratify PBS patients into different management groups (13), the vast range of clinical presentations of PBS also lead to a more phenotype-driven organization. The categories utilized by this system are isolated PBS, PBS-plus, and syndromic PBS. Isolated PBS includes those with the classic triad of abdominal wall musculature deformities, abnormalities of the urinary system, and cryptorchidism. PBS-plus incorporates those with additional anomalies that do not involve the genitourinary system and cannot be attributed to a defined syndrome. Finally, syndromic PBS indicates that the triad of PBS coincides with other known syndromes, such as Duchenne muscular dystrophy, Pierre Robin syndrome, and VACTERL (9).

A multitude of chromosomal abnormalities have been implicated in the pathogenesis of PBS. PBS can occur in association with trisomy 18 and 21 (14–16). Weber et al. performed exon capture and massively parallel sequencing and identified a homozygous frameshift mutation in *CHRM3* gene on chromosome 1q43 (17). Boghossian et al. identified a few copy number variants in patients with PBS; these included a 4q22 duplication that overlaps *BMPR1B* gene, duplications of *STIM1* gene, duplication of *NOG* gene, and a deletion that involved *MYOCD* gene (3). Murray et al. documented the deletion of *HNF-1*β gene on chromosome 17 in a patient with PBS (18). Iqbal et al. reported on PBS patients with mutations of X-linked *filamin A* gene (19).

We report the first documented PBS patient with a copy number variant in 16p11.2. The genetic report showed heterogeneous copy number increase of 839.5 kb involving chromosome 16p11.2. This duplication overlaps the chromosome 16p11.2 deletion syndrome 220-kb critical region (OMIM 613444) and encompasses *SH2B1* gene (OMIM 608937). A list of duplicated genes is displayed in Table 1. Patients with a deletion of this region present with developmental delay, learning disability, behavioral problems, dysmorphology, and obesity; however, the clinical significance of duplication is not clear at this time. One paper by Sampson et al. reported three patients with a 16p11.2 microdeletion. Each of these patients also presented with congenital anomalies of the kidney and urinary tract (CAKUT) as well as Hirschsprung disease (20). This *SH2B1* gene is also associated with leptin signaling in the brain and insulin signaling. Another patient within our medical system is an 8-year-old male with a 16p11.2 microdeletion with a history of Klinefelter syndrome and bilateral retractile testicles. Though these abnormalities are connected to deletion rather than duplication of this chromosomal segment, it does point toward a strong connection between 16p11.2 and urinary development.

**Table 1:** Genes included in the 16p11.2 duplication (20–29).

**Gene**	**Gene information**	**Previously described associations**
*SH2B1*	*SH2B1* codes for Src homology 2B, which has been associated with leptin signaling in the brain, insulin signaling in peripheral tissues, and β-cell expansion in pancreatic islet cells.	Obesity, type 2 diabetes, Hirschsprung disease, congenital anomalies of the kidney and urinary tract (CAKUT)
*CLN3*	Ceroid-lipofuscinosis, neuronal 3 gene codes for a transmembrane protein consisting of 438 amino acids and is widely believed to primarily localize to late endosomal and lysosomal membranes.	Juvenile-onset neuronal ceroid lipofuscinosis
*APOBR*	*APOBR* codes for Apolipoprotein B48, which is a major component of chylomicrons, VLDL, and LDL.	Hypercholesterolemia
*IL27*	*IL-27* codes for a subunit of a cytokine complex that plays a role in expansion of naïve CD4+ cell and the Th-1 pathway.	Unknown
*NUPR1*	Nuclear Protein 1 is a transcriptional regulator that contributes to cells' resistance to stress from changes in their environment. It aids the establishment of metastasis and plays a key role in the progression of several malignancies including those of the breast, thyroid, brain, and pancreas.	For prostate cancer, NUPR1 appears to have tumor suppressive activity.
*SGF29*	SAGA Complex Associated Factor 29 is a protein coding gene involved in the response to endoplasmic reticulum stress by recruiting the SAGA complex to H3K4me and, thereby, promoting histone H3 acetylation and cell survival.	Unknown
*SULT1A1/2*	Sulfotransferase Family 1A Member 2 belongs to a group of enzymes that catalyze the sulfate conjugation of many hormones, neurotransmitters, drugs, and xenobiotic compounds. One study showed this is to be associated with increased risk of breast cancer.	One study showed this is to be associated with increased risk of breast cancer.
*NPIPB8/9*	Nuclear Pore Complex Interacting Protein Family Member B8 is a protein-coding gene that has been shown to be expressed in the testes.	Unknown
*EIF3CL*	Eukaryotic Translation Initiation Factor 3 Subunit C Like binds the 40S ribosome and mRNAs, which allows for translation initiation to occur.	Unknown
*MIR6862-1/2*	MicroRNAs (miRNAs) are short non-coding RNAs that are involved in post-transcriptional gene regulation that impacts both stability and translation of mRNAs.	Unknown
*ATXN2L*	Codes for ataxin type 2-related protein.	Spinocerebellar ataxia 2, Niemann–Pick disease
*MIR4721*	MicroRNAs (miRNAs) are short non-coding RNAs that are involved in post-transcriptional gene regulation that impact both stability and translation of mRNAs.	Retinitis pigmentosa
*TUFM*	TUFM encodes a protein that participates in protein translation in mitochondria.	Combined oxidative phosphorylation deficiency 4
*ATP2A1* and *ATP2A1-AS1*	ATP2A1 codes for sarcoplasmic reticulum calcium-ATPase 1 (SERCA1), an enzyme that regulates calcium ion levels in skeletal muscle cells.	Brody myopathy
*RABEP2*	RABEP2 (Rabaptin, RAB GTPase Binding Effector Protein 2) is a protein-coding gene.	Koolen-De Vries syndrome
*CD19*	The CD19 gene codes for a B-cell coreceptor for that lowers the threshold for triggering B-cell responses.	Common variable immunodeficiency
*MIR4517*	MicroRNAs (miRNAs) are short non-coding RNAs that are involved in post-transcriptional gene regulation that impact both stability and translation of mRNAs.	Unknown
*NFATC2IP*	NFATC2IP (Nuclear Factor of Activated T Cells 2 Interacting Protein) is a protein coding gene. In Th-2 cells, it promotes cytokine production.	Friedreich ataxia 2 and tuberculous empyema
*SPNS1*	SPNS1 (Sphingolipid Transporter 1) is a protein coding gene.	Amelogenesis imperfecta, type Ig

*VLDL, very-low-density lipoprotein; LDL, low-density lipoprotein*.

In this report, we present the first documented case report of a patient with PBS and a concurrent gene duplication of the 16p11.2 gene segment. As no single gene mutation has been identified as the definite cause of PBS, gene analysis of additional patients with this condition is needed to compare this patient's report.

## Data Availability Statement

The original contributions presented in the study are included in the article/supplementary material, further inquiries can be directed to the corresponding author/s.

## Ethics Statement

Written informed consent was obtained from the participants' parents for the publication of this case report.

## Author Contributions

All authors have made significant contributions to the manuscript including design, drafting, revising, and approved the final manuscript and its submission to *Frontiers*.

## Conflict of Interest

The authors declare that the research was conducted in the absence of any commercial or financial relationships that could be construed as a potential conflict of interest.

## Publisher's Note

All claims expressed in this article are solely those of the authors and do not necessarily represent those of their affiliated organizations, or those of the publisher, the editors and the reviewers. Any product that may be evaluated in this article, or claim that may be made by its manufacturer, is not guaranteed or endorsed by the publisher.
